# P-1732. Antifungal Susceptibility Patterns of Major Candida Species Isolated From 1390 Bloodstream Infections in Costa Rica: A 12-Year Experience

**DOI:** 10.1093/ofid/ofaf695.1903

**Published:** 2026-01-11

**Authors:** Juan Villalobos Vindas, Jose A Castro Cordero, Elvira Segura Retana, Carlos Ramírez Valverde, Randall G León Solís, Saúl Quirós Cárdenas, Heylin Estrada Murillo, Alvaro A Aviles Montoya, Laura Villalobos González

**Affiliations:** Caja Costarricense de Seguro Social, San José, San Jose, Costa Rica; Caja Costarricense de Seguro Social, San José, San Jose, Costa Rica; Caja Costarricense de Seguro Social, San José, San Jose, Costa Rica; Caja Costarricense del Seguro Social, San José, San Jose, Costa Rica; Caja Costarricense de Seguro Social, San José, San Jose, Costa Rica; CCSS, San Jose, San Jose, Costa Rica; Caja Costarricense del Seguro Social, San José, San Jose, Costa Rica; Caja Costarricense de Seguro Social, San José, San Jose, Costa Rica; Caja Costarricense de Seguro Social, San José, San Jose, Costa Rica

## Abstract

**Background:**

*Candida* species represent 80% of nosocomial fungal infections, with candidemia being the most frequent invasive disease. In Latin America, *Candida* resistance is generally low, but information from Central America is scarce. We aimed to describe antifungal susceptibility of *Candida* species isolated from bloodstream infections at two national adult hospitals in Costa Rica from 2012 to 2023.

**Methods:**

This retrospective study analyzed the first isolate from candidemia episodes caused by *C. albicans*, *C. tropicalis*, *C. parapsilosis*, and *C. glabrata* with available susceptibility results. Testing used VITEK 2 system. Interpretation followed CLSI M27M44S-Ed3 guidelines. Amphotericin B susceptibility was defined as MIC ≤1μg/ml. Differences between species were assessed using Fisher's exact test, and temporal trends with chi-square test.

**Results:**

We analyzed 1,390 isolates: *C. parapsilosis* 689 (49.6%), *C. albicans* 448 (32.2%), *C. tropicalis* 131 (9.4%), and *C. glabrata* 122 (8.8%). Fluconazole susceptibility was high in *C. albicans* (97.1%) and *C. tropicalis* (96.2%), but lower in *C. parapsilosis* (40.6%, p< 0.001). *C. glabrata* isolates were predominantly susceptible-dose dependent (96.1%). Voriconazole showed high activity against *C. albicans* (98.7%) and *C. tropicalis* (96.9%), but resistance in *C. parapsilosis* (20.2%). Amphotericin B maintained excellent activity against all species (98.7% overall). Echinocandins demonstrated high efficacy against *C. albicans*, *C. tropicalis*, and *C. parapsilosis* (≥99%), but reduced activity against *C. glabrata* (caspofungin 41.3% susceptible, micafungin with 70.6% intermediate and 29.4% resistant). No significant temporal trends in susceptibility were observed over the 12-year period.

**Conclusion:**

Our findings reveal significant azole resistance in *C. parapsilosis*, particularly to fluconazole (59.4%) and voriconazole (20.2%). Amphotericin B maintains excellent activity against all species. Echinocandins remain highly effective against most *Candida* species except *C. glabrata*. These patterns have important implications for empirical antifungal therapy selection in our region.

**Disclosures:**

All Authors: No reported disclosures

